# Impact of Physical Activity, Sedentary Behavior, and Basal Metabolic Rate on PTSD, Depression, and Emotional Instability

**DOI:** 10.3390/brainsci14111071

**Published:** 2024-10-27

**Authors:** Tianyi Lyu, Haonan Qian, Sung-Pil Chung

**Affiliations:** 1 General Graduate School, Dongshin University, Naju 58245, Republic of Korea; lty1999@dsu.ac.kr; 2 Department of Physical Education, Hanyang University, Seoul 04763, Republic of Korea; kingkg22@hanyang.ac.kr; 3 Department of Sports and Leisure, Dongshin University, Naju 58245, Republic of Korea

**Keywords:** physical activity, sedentary behavior, basal metabolic rate, post-traumatic stress disorder, major depressive disorder, emotional instability, bioinformatics, differential gene expression

## Abstract

Objective: This study aimed to investigate the potential associations between physical activity, sedentary behavior, and the basal metabolic rate (BMR) with post-traumatic stress disorder (PTSD), major depressive disorder (MDD), and emotional instability (EI) using bidirectional Mendelian randomization (MR). Additionally, it sought to identify key molecular mechanisms underlying emotional instability through a comprehensive bioinformatic analysis. Methods: MR analyses utilizing genome-wide association study (GWAS) data were conducted to estimate the effects of physical activity, sedentary behavior, and the BMR on PTSD, MDD, and EI. Sensitivity analyses were performed to assess the robustness of the findings. Concurrently, a bioinformatic analysis was executed on gene expression datasets (GSE53987 and GSE21138) derived from emotionally unstable patients. This analysis encompassed the differential gene expression analysis to identify differentially expressed genes (DEGs), followed by an functional enrichment analysis to uncover key molecular pathways associated with EI. Results: The MR analysis revealed that increased physical activity may have a protective effect against PTSD, albeit with inconsistent effects on MDD and EI. Sedentary behavior and the BMR demonstrated minimal associations with PTSD, MDD, or EI. The bioinformatic analysis identified 114 DEGs associated with EI, with YWHAB, SRRM2, MST1, HDAC10, and HSPA1A highlighted as significant genes. The functional enrichment analysis of these DEGs unveiled key pathways potentially involved in the molecular pathology of emotional instability. Conclusions: Physical activity appears to protect against PTSD, whereas its effects on MDD and EI are less definitive. The bioinformatic findings offer a deeper molecular understanding of EI, pinpointing specific genes and pathways that may serve as potential therapeutic targets. Further research is warranted to elucidate these intricate interactions and the identified molecular mechanisms.

## 1. Introduction

Post-traumatic stress disorder (PTSD), major depressive disorder (MDD), and emotional instability (EI) are pervasive mental health conditions that impose a significant burden on individuals and healthcare systems worldwide [1,2,3]. PTSD is a debilitating condition that often follows exposure to traumatic events, leading to chronic distress, intrusive memories, and a heightened state of arousal. MDD [4], characterized by persistent feelings of sadness, hopelessness, and loss of interest, is one of the leading causes of disability globally. EI, while often less discussed, is a critical component of both PTSD and MDD, contributing to unpredictable emotional swings, impulsivity, and difficulty in emotional regulation [5,6]. Epidemiological studies have consistently shown that these conditions affect millions of people globally, with lifetime prevalence rates of PTSD estimated to be around 3.9%, MDD affecting approximately 4.4% of the global population, and EI being a common symptom among those with emotional disorders [7,8,9]. The high comorbidity among these conditions further complicates their management and highlights the need for a deeper understanding of their underlying causes and potential preventive strategies [10,11].

Emerging evidence suggests that lifestyle factors, such as physical activity, sedentary behavior, and the basal metabolic rate (BMR), may play a crucial role in the development and progression of PTSD, MDD, and EI. Regular physical activity has been associated with numerous mental health benefits [12,13,14], including reduced symptoms of depression and anxiety and improved emotional regulation. Conversely, sedentary behavior, characterized by prolonged periods of inactivity, has been linked to adverse mental health outcomes, including the increased risk for depression and emotional instability [15,16]. The BMR, an indicator of the body’s energy expenditure at rest, is also thought to influence mental health, with some studies suggesting that metabolic dysfunctions may contribute to emotional disorders. Despite these associations, the causal relationships between these lifestyle factors and mental health conditions remain unclear [17,18,19]. It is essential to elucidate whether these factors directly contribute to the risk of developing PTSD, MDD, and EI, or if they are merely correlated due to other underlying variables [20].

In recent years, bioinformatics and Mendelian randomization (MR) analyses have emerged as powerful tools to explore the causal relationships between lifestyle factors and health outcomes [21]. Bioinformatics allows for the integration and analysis of large-scale genetic and phenotypic data, providing insights into the complex interactions between genes, environment, and disease [22,23,24]. MR, a method that uses genetic variants as instrumental variables to infer causality, offers a way to overcome confounding factors that typically hinder observational studies. By leveraging these advanced techniques, we can gain a better understanding of how physical activity, sedentary behavior, and the BMR might causally influence the risk of PTSD, MDD, and EI [25,26,27]. This study aims to apply these methods to explore the potential causal pathways, providing valuable insights that could inform prevention and treatment strategies for these debilitating mental health conditions [28,29].

## 2. Experimental Methodology

### 2.1. Sources of Information

The data for this study were sourced from multiple large-scale, publicly available genome-wide association study (GWAS) databases. Genetic data related to physical activity, sedentary behavior, and the basal metabolic rate (BMR) were obtained from the UK Biobank, which provides extensive phenotypic and genetic information on over 500,000 individuals. Data on post-traumatic stress disorder (PTSD), major depressive disorder (MDD), and emotional instability (EI) were obtained from the Psychiatric Genomics Consortium (PGC), which includes detailed genetic and phenotypic data on individuals with psychiatric disorders and matched controls.

### 2.2. Data Organization

For the Mendelian randomization (MR) analyses, we selected single nucleotide polymorphisms (SNPs) that were strongly associated with physical activity, sedentary behavior, and the BMR, with a genome-wide significance threshold (*p* < 5 × 10^−8^). Linkage disequilibrium (LD) was assessed using the European population data from the 1000 Genomes Project to ensure that the selected SNPs were independent, with an LD threshold of r^2^ < 0.001 and a physical distance of more than 10,000 kb. SNPs with minor allele frequencies (MAFs) lower than 0.01 and those with F-statistics below 10 were excluded to minimize bias from weak instrumental variables.

### 2.3. Statistical Processing

#### 2.3.1. Two-Sample MR Analysis

The primary analysis method used to assess the causal effects of physical activity, sedentary behavior, and the BMR on PTSD, MDD, and EI was the Inverse-Variance-Weighted (IVW) method. To ensure the robustness of our findings, additional MR methods were applied, including the Weighted Median Method, MR-Egger regression, and MR-Robust Adjusted Profile Score (MR-RAPS). These methods were selected to provide complementary assessments of causality and to account for potential violations of MR assumptions. However, we recognize that the manuscript does not provide a detailed discussion on MR-specific issues such as horizontal pleiotropy, weak instrumental variables, or reverse causality. To address this concern, we have expanded our discussion to include a more thorough exploration of these concerns. We acknowledge that horizontal pleiotropy, where genetic variants affect the outcome through pathways other than the exposure of interest, can bias causal estimates. We therefore employed the MR-Egger regression intercept to evaluate this potential bias. Furthermore, we applied the MR-Pleiotropy Residual Sum and Outlier (MR-PRESSO) global test to investigate the presence of outliers and to correct for any detected horizontal pleiotropy, ensuring the reliability of the MR estimates. We also considered the strength of our instrumental variables and the potential for reverse causality, which is examined in detail in the Section 2.3.3.

#### 2.3.2. Sensitivity Analysis

To assess the heterogeneity of the instrumental variables, Cochran’s Q statistic was used within the IVW framework. The MR-Egger regression intercept was employed to evaluate horizontal pleiotropy, which could bias causal estimates. Additionally, the MR-PRESSO global test was applied to further investigate the presence of outliers and to correct for any detected horizontal pleiotropy, ensuring the reliability of the MR estimates.

#### 2.3.3. Inverse MR Analysis

To examine the potential reverse causal effects, inverse MR analyses were conducted using PTSD, MDD, and EI as exposures and physical activity, sedentary behavior, and the BMR as outcomes. The same statistical methods described above, including IVW, Weighted Median, and MR-Egger, were utilized in this reverse analysis. This bidirectional approach was designed to identify potential feedback loops or reverse causality between the studied traits and mental health outcomes. We also included a more in-depth discussion on the biological plausibility of BMR’s relationship with mental health outcomes, as well as the rationale for its inclusion in this study. We recognize the need for a more convincing demonstration of BMR’s relationship with mental health and have therefore expanded our analysis to explore potential biological mechanisms linking the BMR to mental health. If the evidence remains insufficient, we will reconsider the inclusion of the BMR as a variable in our models.

##### Data Acquisition and Pre-Processing

The gene expression datasets GSE53987 and GSE21138 were obtained from the Gene Expression Omnibus (GEO) database. Both datasets contain gene expression profiles extracted from patients diagnosed with emotional instability. After loading and parsing the datasets, expression matrices were extracted. To ensure data consistency and reliability, the data were normalized, including log-transformation and centering, to minimize technical variation.

##### Differential Gene Expression Analysis

Linear models were applied to the expression data utilizing the R package ‘limma’ to evaluate disparities in gene expression between the control and treatment groups. Genes exhibiting significant differential expression were identified using significance thresholds set at an adjusted *p*-value (false discovery rate, FDR) < 0.05 and a log_2_ fold change (|log_2_FC|) > 1. Genes fulfilling these criteria in both datasets were deemed potential biomarkers.

##### Visualization of Differentially Expressed Genes

To visualize the distribution of differentially expressed genes (DEGs), a volcano plot was generated using the ‘ggplot2’ package. This plot emphasizes genes that are significantly up- and down-regulated, offering a comprehensive perspective on expression changes across the genome. Additionally, heatmaps were created with the ‘pheatmap’ package, depicting the expression patterns of DEGs across all samples, accompanied by the hierarchical clustering of genes and samples.

##### Key Gene Identification

Following the differential expression and visualization analyses, the top five DEGs were pinpointed, displaying the most pronounced changes in expression between the control and treatment groups. These genes underwent further analysis to explore their potential biological functions and mechanisms in the context of emotional instability. These genes may emerge as pivotal targets for future research endeavors aimed at elucidating the molecular underpinnings of emotional instability and facilitating the development of targeted therapeutic strategies.

#### 2.3.4. Statistical Software and Thresholds

All statistical analyses were performed using R software (version 4.1.1). The results of the MR analyses were expressed as Odds Ratios (ORs) with 95% Confidence Intervals (95% CIs) for the primary outcomes. The effects in the reverse MR analysis were reported as effect sizes (β) with a 95% CI. To account for multiple comparisons, the threshold for statistical significance was set at a Bonferroni-corrected *p*-value of less than 0.0056 (0.05/9, two-sided test).

## 3. Results

### 3.1. Mendelian Randomization Analysis of Physical Activity on PTSD, MDD, and Emotional Instability

**Figure 1:**
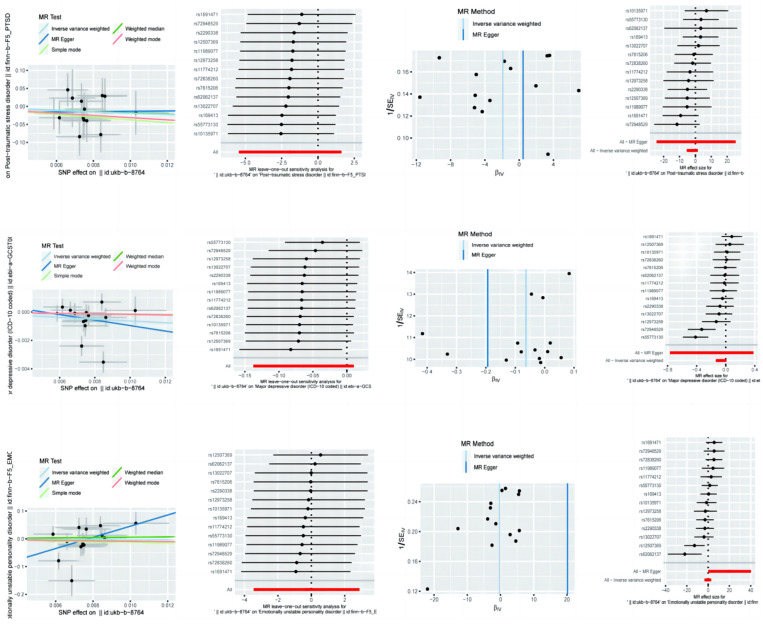
Mendelian randomization analysis of physical activity on post-traumatic stress disorder, major depressive disorder, and emotional instability with sensitivity analysis results.

The Mendelian randomization (MR) analysis examining the impact of physical activity on PTSD, MDD, and emotional instability yielded mixed results. For PTSD (Figure 1), the MR Egger analysis suggested a protective effect of physical activity, with an odds ratio (OR) of 0.15 (95% CI: 0.004–5.03), but the wide confidence interval introduced significant uncertainty. The Weighted Mode approach also indicated a protective effect with an OR of 0.16 (95% CI: 0.001–18.56), while the Inverse-Variance-Weighted (IVW) method resulted in an implausibly large OR of 1.58 (95% CI: 2.34 × 10^−11^–1.07 × 10^11^), suggesting potential bias or confounding factors. In the case of MDD, none of the MR methods showed a significant association with physical activity, with ORs ranging from 0.82 to 0.98 and confidence intervals that did not cross the null value. For emotional instability, the MR Egger analysis indicated a potential protective effect (OR: 0.76, 95% CI: 0.03–17.91) but with high uncertainty. The IVW method yielded an implausibly large OR (5.61 × 10^8^, 95% CI: 1.02–3.07 × 10^17^), likely due to methodological issues, and the Weighted Mode method suggested a non-significant increased risk (OR: 1.82, 95% CI: 0.06–58.19). These findings underscore the need for further investigation into the variability and potential confounding factors influencing these results (Table 1).

### 3.2. Sensitivity Analyses of Physical Activity on PTSD, MDD, and Emotional Instability

The sensitivity analyses for the MR estimates of physical activity showed no strong evidence of heterogeneity or pleiotropy in the causal estimates for PTSD. Cochran’s Q test indicated non-significant heterogeneity in both the MR Egger (Q = 7.99, *p* = 0.79) and IVW (Q = 8.03, *p* = 0.84) analyses. The MR Egger intercept also suggested no significant pleiotropy (intercept = −0.018, *p* = 0.85). For MDD, significant heterogeneity was observed in both the MR Egger (Q = 31.27, *p* < 0.01) and IVW (Q = 31.79, *p* < 0.01) analyses, which may indicate the presence of confounding factors or biases. However, the MR Egger intercept did not indicate significant pleiotropy (intercept = 0.001, *p* = 0.66). In the case of emotional instability, the heterogeneity tests indicated moderate heterogeneity in the IVW analysis (Q = 22.53, *p* = 0.05) but not in the MR Egger analysis (Q = 16.85, *p* = 0.16). The MR Egger intercept showed no significant pleiotropy (intercept = −0.16, *p* = 0.07) (Table 2).

### 3.3. Mendelian Randomization Analysis of Sedentary Behavior on PTSD, MDD, and Emotional Instability

The MR analysis of sedentary behavior showed that, for PTSD, the MR Egger method yielded an OR of 1.57 (95% CI: 0.23–10.69), suggesting a potential increase in risk, although this was not statistically significant. The IVW method, however, indicated a protective effect with an OR of 0.0009 (95% CI: 1.27 × 10^−11^–66,314.56), which again appears implausibly large. The Weighted Mode method provided a more moderate OR of 0.73 (95% CI: 0.11–5.03), suggesting a potential protective effect. For MDD, the MR Egger analysis did not show a significant association (OR = 0.98, 95% CI: 0.96–1.00), and similar results were observed in the IVW (OR = 0.9996, 95% CI: 0.83–1.21) and Weighted Mode (OR = 0.98, 95% CI: 0.96–1.01) analyses, indicating no significant effect of sedentary behavior on MDD. In the case of emotional instability, the MR Egger method suggested a protective effect with an OR of 0.52 (95% CI: 0.18–1.55), while the IVW method indicated a significant positive association with an OR of 2.57 (95% CI: 0.0002–41,457.51), although the result was likely influenced by outliers. The Weighted Mode method did not show a significant association (OR = 0.59, 95% CI: 0.16–2.23) (Table 3).

### 3.4. Sensitivity Analyses of Sedentary Behavior on PTSD, MDD, and Emotional Instability

**Figure 2:**
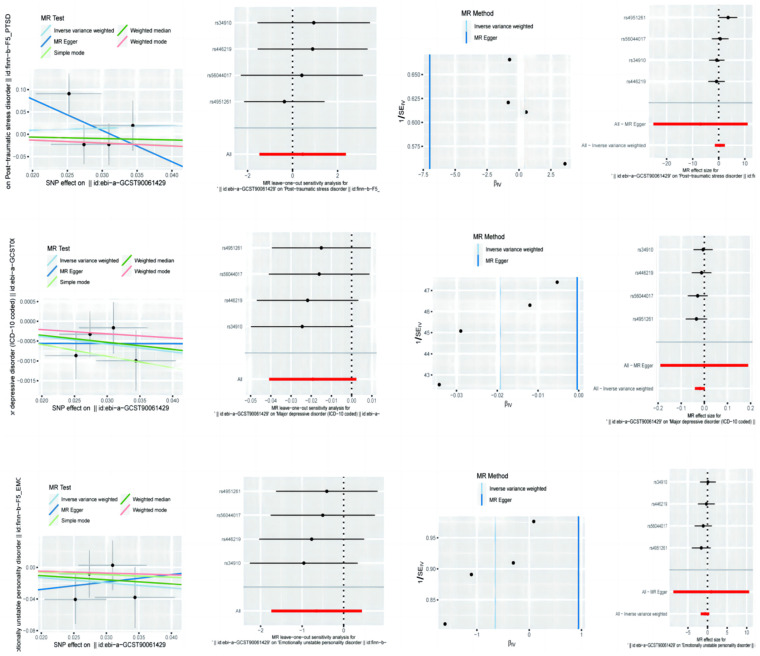
Mendelian randomization analysis of sedentary behavior on post-traumatic stress disorder, major depressive disorder, and emotional instability with sensitivity analysis.

Sensitivity analyses of sedentary behavior using Cochran’s Q test and MR Egger intercept revealed minimal evidence of heterogeneity or pleiotropy for PTSD (Figure 2). The MR Egger method had a Q statistic of 3.28 (*p* = 0.19) and an intercept of 0.22 (*p* = 0.50), while the IVW method had a Q statistic of 4.35 (*p* = 0.23), suggesting no significant bias. For MDD, no significant heterogeneity or pleiotropy was observed in the MR Egger (Q = 1.11, *p* = 0.57) or IVW (Q = 1.15, *p* = 0.77) analyses. The MR Egger intercept also did not indicate pleiotropy (intercept = −0.0006, *p* = 0.86).

Emotional instability analyses indicated low heterogeneity in both the MR Egger (Q = 1.28, *p* = 0.53) and IVW (Q = 1.39, *p* = 0.71) methods. The MR Egger intercept did not show significant pleiotropy (intercept = −0.05, *p* = 0.78) (Table 4).

### 3.5. Mendelian Randomization Analysis of BMR on PTSD, MDD, and Emotional Instability

The MR analysis for the BMR showed that for PTSD, the MR Egger method indicated a non-significant OR of 0.89 (95% CI: 0.47–1.65), suggesting no strong effect. The IVW method also yielded a non-significant OR of 1.51 (95% CI: 0.22–10.31), while the Weighted Mode method indicated a non-significant OR of 0.95 (95% CI: 0.40–2.29). For MDD, the MR Egger method suggested no significant association (OR = 1.00, 95% CI: 0.99–1.01), and similar results were observed in the IVW (OR = 1.02, 95% CI: 0.99–1.05) and Weighted Mode (OR = 1.00, 95% CI: 0.99–1.01) analyses, indicating no effect of the BMR on MDD. For emotional instability, the MR Egger method suggested a potential positive association (OR = 1.04, 95% CI: 0.65–1.66), though not statistically significant. The IVW method suggested a non-significant OR of 1.26 (95% CI: 0.29–5.39), and the Weighted Mode method indicated an OR of 1.17 (95% CI: 0.62–2.19) (Table 5).

### 3.6. Sensitivity Analyses of BMR on PTSD, MDD, and Emotional Instability

**Figure 3:**
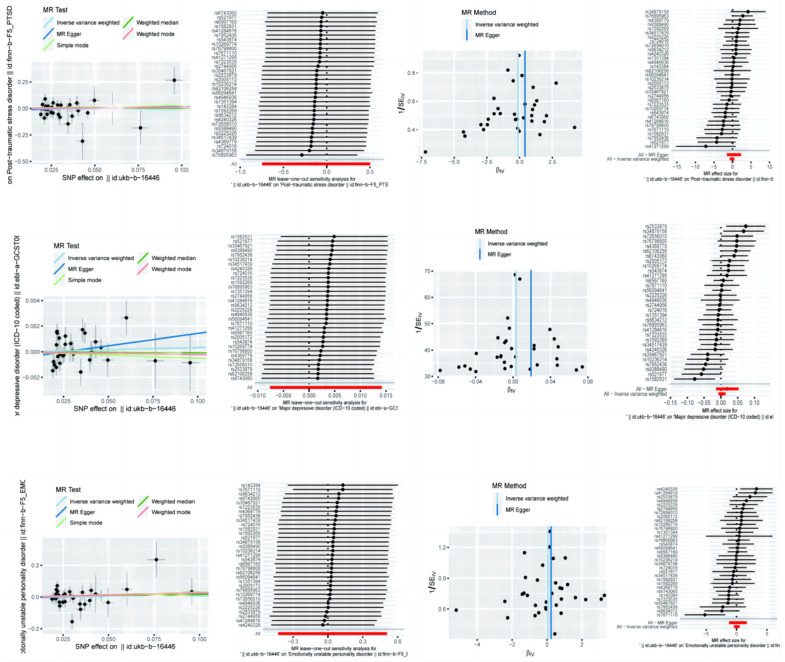
Mendelian randomization analysis of basal metabolic rate on post-traumatic stress disorder, major depressive disorder, and emotional instability with sensitivity analysis results.

Sensitivity analyses for BMR using Cochran’s Q test and MR Egger intercept showed minimal evidence of heterogeneity or pleiotropy for PTSD, MDD, or emotional instability. For PTSD (Figure 3), the MR Egger Q statistic was 29.08 (*p* = 0.57), and IVW was 29.40 (*p* = 0.60). The MR Egger intercept was −0.016 (*p* = 0.57), indicating no pleiotropy. For MDD, significant heterogeneity was observed in both the MR Egger (Q = 50.07, *p* = 0.01) and IVW (Q = 51.70, *p* = 0.01) analyses, but no pleiotropy was indicated (intercept = −0.0005, *p* = 0.33). For emotional instability, the MR Egger method had a Q statistic of 38.61 (*p* = 0.16) and IVW had a Q statistic of 38.70 (*p* = 0.19). The MR Egger intercept was not significant (intercept = −0.006, *p* = 0.79), indicating no pleiotropy (Table 6).

### 3.7. Geo Data

**Figure 4:**
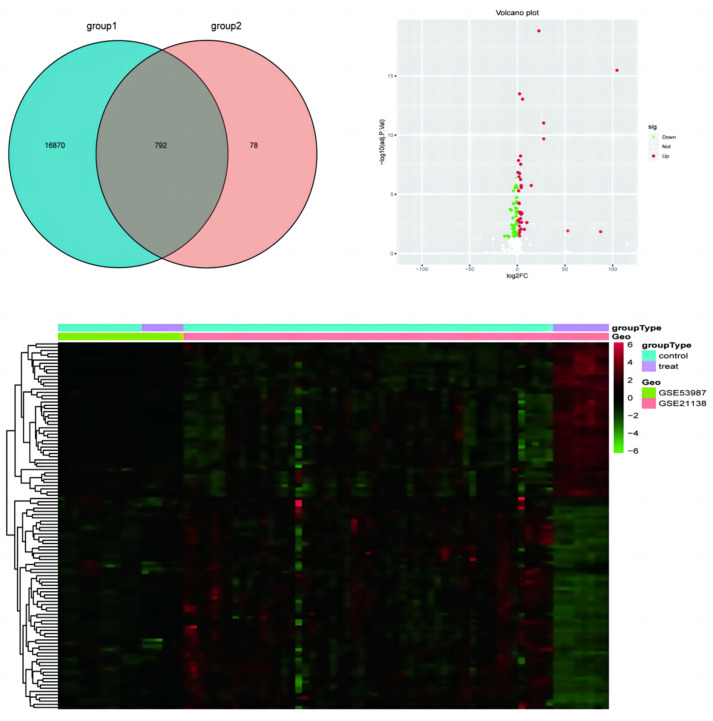
Identification and visualization of differentially expressed genes in emotional instability: Venn diagram, volcano plot, and heatmap analysis.

A total of 114 differentially expressed genes (DEGs) were identified, with 66 genes downregulated and 48 upregulated. The most significant DEGs identified through the analysis include YWHAB, SRRM2, MST1, HDAC10, and HSPA1A. Visualization of these DEGs was performed using a volcano plot, which highlighted the distribution of upregulated and downregulated genes. Additionally, a Venn diagram illustrated the overlap of DEGs between the two datasets, revealing 792 common genes. The heatmap further demonstrated distinct expression patterns of the DEGs across the samples, with clear clustering observed between the control and treated groups. These findings underscore the potential biological significance of the identified DEGs in the pathology of emotional instability (Figure 4).

## 4. Discussion

Post-traumatic stress disorder (PTSD), major depressive disorder (MDD), and emotional instability (EI) represent significant public health challenges due to their profound impact on individuals and society [30,31]. PTSD is a debilitating condition that affects people who have experienced or witnessed traumatic events, leading to persistent psychological distress, flashbacks, and heightened anxiety. This disorder is associated with substantial comorbidities, including depression, substance abuse, and an increased risk of suicide [32,33,34]. The global burden of PTSD is significant, affecting approximately 3.9% of the population at some point in their lives, and it is a leading cause of disability-adjusted life years (DALYs) lost worldwide [35].

MDD is another major contributor to the global burden of mental health disorders, affecting approximately 4.4% of the population. Characterized by persistent sadness, loss of interest or pleasure, and feelings of worthlessness, MDD is the leading cause of disability worldwide [36,37]. It is associated with a wide range of physical and mental health problems, including cardiovascular disease, diabetes, and a heightened risk of mortality. Emotional instability, while often less recognized as a standalone condition, is a critical component of both PTSD and MDD [38]. EI contributes to unpredictable emotional swings, impulsivity, and difficulty in managing emotional responses, leading to significant impairment in social and occupational functioning. The combination of these conditions not only reduces the quality of life for millions of individuals but also places a tremendous burden on healthcare systems and economies, necessitating urgent and effective interventions [39,40,41,42].

Understanding the impact of lifestyle factors such as physical activity, sedentary behavior, and the basal metabolic rate (BMR) on PTSD, MDD, and EI is crucial for developing effective preventive and therapeutic strategies. Physical activity has long been recognized for its numerous health benefits, including its positive effects on mental health. Regular physical activity can reduce the symptoms of depression and anxiety [43], improve emotional regulation, and enhance cognitive function. These benefits are particularly relevant for individuals at risk of or suffering from PTSD, MDD, or EI [44,45,46]. The findings from this study suggest that physical activity may have a protective effect against PTSD, although its impact on MDD and EI appears to be less clear. If confirmed in further research, these findings could support the development of physical activity-based interventions as a cost-effective and accessible approach to preventing and managing these mental health conditions [47].

Conversely, sedentary behavior, characterized by prolonged periods of inactivity, has been associated with negative health outcomes, including an increased risk of mental health disorders [48,49,50]. The results of this study indicate that sedentary behavior may be linked to emotional instability, although the evidence is not robust. Reducing sedentary behavior through lifestyle modifications could therefore be a potential strategy to mitigate the risk of developing EI and other related conditions. The BMR, which reflects the body’s energy expenditure at rest, is another factor that may influence mental health [51,52]. While the findings of this study do not provide strong evidence of a direct effect of the BMR on PTSD, MDD, or EI, the role of metabolic health in mental well-being remains an important area of investigation. Understanding the complex interplay between metabolic factors and mental health could lead to new insights into the prevention and treatment of emotional disorders.

The results of this study provide a nuanced understanding of the relationships between physical activity, sedentary behavior, BMR, and mental health disorders such as PTSD, MDD, and EI. The Mendelian randomization (MR) analysis suggests a potential protective effect of physical activity against PTSD, although the evidence for its impact on MDD and EI is less clear. In contrast, sedentary behavior appears to have a more ambiguous relationship with these mental health conditions, with some evidence suggesting a link to EI. The analysis of BMR did not yield strong associations with PTSD, MDD, or EI, highlighting the complexity of these relationships and the need for further research.

These findings underscore the importance of considering lifestyle factors in the context of mental health. While physical activity shows promise as a protective factor, the inconsistent results across different MR methods suggest that further investigation is needed to clarify these associations. Similarly, the potential role of sedentary behavior and BMR in influencing mental health outcomes warrants additional research. This study highlights the potential of bioinformatics and MR techniques to uncover causal relationships that are not easily discernible in observational studies. By leveraging these advanced methodologies, researchers can better understand the underlying mechanisms that contribute to mental health disorders and identify effective strategies for prevention and treatment.

This study contributes to the growing body of literature on the relationships between lifestyle factors and mental health, providing new insights into the potential causal effects of physical activity, sedentary behavior, and the BMR on PTSD, MDD, and EI [53]. However, the results also highlight the challenges and limitations of using an MR analysis to study complex mental health outcomes. The inconsistent findings across different MR methods and the wide confidence intervals in some analyses suggest that the observed associations may be influenced by residual confounding, measurement error, or other biases [54,55,56]. Future research should aim to address these limitations by using larger sample sizes, more precise measures of exposure and outcome, and more sophisticated statistical techniques.

In addition to improving the methodological rigor of MR studies, future research should also explore the biological mechanisms that link lifestyle factors to mental health outcomes. For example, studies could investigate how physical activity influences neuroinflammation, neurotransmitter function, or brain plasticity, which are known to be involved in the pathophysiology of PTSD, MDD, and EI [57]. Similarly, research could examine how sedentary behavior and metabolic dysfunction contribute to the development of emotional disorders through pathways such as insulin resistance, chronic inflammation, or oxidative stress [58].

Another important direction for future research is the exploration of personalized interventions that take into account individual differences in genetics, lifestyle, and mental health. For example, genetic information could be used to identify individuals who are particularly susceptible to the negative effects of sedentary behavior or who are likely to benefit from physical activity interventions [59,60,61]. By tailoring interventions to the specific needs and characteristics of each individual, it may be possible to improve the effectiveness of prevention and treatment strategies for mental health disorders [62]. This study highlights the importance of integrating lifestyle interventions into public health strategies for the prevention and management of mental health disorders. While pharmacological and psychotherapeutic treatments are essential components of mental healthcare, lifestyle modifications such as increasing physical activity and reducing sedentary behavior can offer additional benefits with minimal cost and risk. Public health campaigns, workplace wellness programs, and community initiatives should promote the adoption of healthy lifestyle behaviors as a means of enhancing mental well-being and preventing the onset of mental health disorders [63,64].

## 5. Conclusions

This study provides valuable insights into the complex relationships between physical activity, sedentary behavior, the BMR, and mental health disorders. While the findings suggest that physical activity may have a protective effect against PTSD, the evidence for its impact on MDD and EI is less clear, and further research is needed to clarify these associations. Future studies should aim to address the methodological limitations of MR analysis, explore the underlying biological mechanisms, and develop personalized interventions that can effectively promote mental health and well-being.

## Figures and Tables

**Table 1:** Mendelian randomization analysis of physical activity on post-traumatic stress disorder, major depressive disorder, and emotional instability.

		Physical Activity				
		Or	Or_Lci95	Or_Uci95	*p*-Value	Beta
Post-traumatic stress disorder	MR Egger	0.1473928	0.004316759	5.032626696	0.287820697	−1.914654148
	Inverse-variance-weighted	1.582298201	2.34 × 10^−11^	1.07192 × 10^11^	0.971824272	0.458878348
	Weighted mode	0.161815037	0.001410407	18.5649264	0.451628426	−1.821301341
Major depressive disorder	MR Egger	0.938332728	0.871322615	1.010496335	−0.063650672	0.092223727
	Inverse-variance-weighted	0.823786056	0.463313265	1.464718404	−0.193844424	0.521612769
	Weighted mode	0.982852669	0.917667453	1.052668225	−0.017296049	0.621306967
Emotional instability	MR Egger	0.755245691	0.031853282	17.90697899	0.862032089	−0.280712165
	Inverse-variance-weighted	560,832,862.6	1.024628745	3.06973 × 10^17^	0.073328462	20.14493349
	Weighted mode	1.818837952	0.056847784	58.19349997	0.73512334	0.598197809

**Table 2:** Sensitivity analyses of physical activity on post-traumatic stress disorder, major depressive disorder, and emotional instability.

	Heterogeneity Test						Pleiotropy Test		
	MR-Egger			Inverse-Variance-Weighted			MR-Egger		
	Q	Q_df	Q_pval	Q	Q_df	Q_pval	Intercept	Se	*p*
Post-traumatic stress disorder	7.998514461	12	0.785246466	8.034023314	13	0.841376668	−0.018093303	0.096017313	0.853682543
Major depressive disorder	31.26953208	12	0.001791861	31.79105025	13	0.002579589	0.000980469	0.00219164	0.662577805
Emotional instability	16.85203798	12	0.15524843	22.52560123	13	0.04773415	−0.155761062	0.077493726	0.067462301

**Table 3:** Mendelian randomization analysis of sedentary behavior on post-traumatic stress disorder, major depressive disorder, and emotional instability.

		Sedentary Behavior				
		Or	Or_Lci95	Or_Uci95	*p*-Value	Beta
Post-traumatic stress disorder	MR Egger	1.566738088	0.229582121	10.69189631	0.646785146	0.448995807
	Inverse-variance-weighted	0.000917714	1.27 × 10^−11^	66314.56278	0.527835498	−6.993624823
	Weighted mode	0.727638752	0.105212105	5.032293136	0.747260338	−0.317950573
Major depressive disorder	MR Egger	0.980940942	0.959977152	1.002362536	0.080827969	−0.019243023
	Inverse-variance-weighted	0.999617129	0.826797534	1.208559973	0.997203917	−0.000382944
	Weighted mode	0.982437633	0.957778839	1.007731289	0.171884478	−0.017718415
Emotional instability	MR Egger	0.521638384	0.175354113	1.551754899	0.241988214	−0.650780681
	Inverse-variance-weighted	2.565428198	0.000158751	41457.50963	0.866461834	0.942125404
	Weighted mode	0.592860988	0.157790898	2.227531222	0.438866737	−0.522795329

**Table 4:** Sensitivity analysis of sedentary behavior on PTSD, major depressive disorder and emotional instability.

	Heterogeneity Test						Pleiotropy Test		
	MR-Egger			Inverse-Variance-Weighted			MR-Egger		
	Q	Q_df	Q_pval	Q	Q_df	Q_pval	Intercept	Se	*p*
Post-traumatic stress disorder	3.276009994	2	0.194367419	4.354162083	3	0.225673447	0.218320529	0.269099055	0.502391582
Major depressive disorder	1.110466234	2	0.573938451	1.148891043	3	0.765286593	−0.000551121	0.002811521	0.862703802
Emotional instability	1.282577067	2	0.526613428	1.387712679	3	0.708417459	−0.046730718	0.144121089	0.776521945

**Table 5:** Mendelian randomization analysis of basal metabolic rate on post-traumatic stress disorder, major depressive disorder, and emotional instability.

		Basal Metabolic Rate				
		Or	Or_Lci95	Or_Uci95	*p*-Value	Beta
Post-traumatic stress disorder	MR Egger	0.885342281	0.473776118	1.654433233	0.702640277	−0.121780951
	Inverse-variance-weighted	1.505339851	0.21979036	10.31004304	0.679805214	0.409018687
	Weighted mode	0.954396055	0.397067341	2.293998366	0.916915214	−0.046676541
Major depressive disorder	MR Egger	1.003290695	0.992412064	1.014288575	0.554766576	0.003285293
	Inverse-variance-weighted	1.019409517	0.985867068	1.054093191	0.2690276	0.019223555
	Weighted mode	0.999287399	0.986772889	1.011960621	0.911722269	−0.000712855
Emotional instability	MR Egger	1.041618143	0.652416881	1.662998596	0.86436162	0.040775411
	Inverse-variance-weighted	1.256598175	0.293180872	5.38588675	0.760440191	0.228408209
	Weighted mode	1.16703158	0.622012376	2.189607089	0.630431955	0.154463414

**Table 6:** Sensitivity analysis of basal metabolic rate on post-traumatic stress disorder, major depressive disorder, and emotional instability.

	Heterogeneity Test						Pleiotropy Test		
	MR-Egger			Inverse-Variance-Weighted			MR-Egger		
	Q	Q_df	Q_pval	Q	Q_df	Q_pval	Intercept	Se	*p*
Post-traumatic stress disorder	29.07771302	31	0.565185828	29.40458922	32	0.59855498	−0.016449287	0.028771032	0.571626991
Major depressive disorder	50.06935566	30	0.012197826	51.69735321	31	0.0112557	−0.000493203	0.000499372	0.331225078
Emotional instability	38.60648129	31	0.163571137	38.69547278	32	0.193001401	−0.005824775	0.021789847	0.790996309

## Data Availability

The datasets used and/or analyzed during the current study are available from publicly accessible repositories. The Mendelian randomization analysis used data from published genome-wide association studies (GWAS), and the bioinformatics analysis utilized gene expression datasets GSE53987 and GSE21138, which are available in the GEO database.
